# The causal relationship between antihypertensive drugs and knee osteoarthritis: A drug target Mendelian randomization study

**DOI:** 10.1097/MD.0000000000045328

**Published:** 2025-10-17

**Authors:** Zhixin Li, Yuhao Si, Mengmin Liu, Pengcheng Tu, Yafeng Zhang, Yong Ma, Yang Guo

**Affiliations:** a Institute of Traumatology, New Technology on Trauma Repairment and Reconstruction Laboratory, Nanjing, China; b The First School of Clinical Medicine, Nanjing University of Chinese Medicine, Nanjing, China; c School of Acupuncture-Moxibustion and Tuina, School of Regimen and Rehabilitation, Nanjing University of Chinese Medicine, Nanjing, China; d School of Integrated Chinese and Western Medicine, Nanjing University of Chinese Medicine, Nanjing, China; e Jiangsu CM Clinical Innovation Center of Degenerative Bone & Joint Disease, Wuxi TCM Hospital Affiliated to Nanjing University of Chinese Medicine, Wuxi, China; f Yancheng TCM Hospital Affiliated to Nanjing University of Chinese Medicine, Yancheng, China.

**Keywords:** hypertension, knee osteoarthritis, loop diuretics, Mendelian randomization analysis

## Abstract

Recently studies have revealed a robust association between hypertension and knee osteoarthritis (KOA), with patients likely to suffer from both conditions. We employed Mendelian randomization (MR) analysis to assess the impact of antihypertensive medications on KOA, aiming to offer clinical guidance for concomitant drug therapy and identify potential therapeutic targets for KOA. We obtained exposure instruments (instrumental variables) by locating Single-nucleotide polymorphisms related to systolic blood pressure near drug target genes. We then conducted Mendelian randomization analyses between the exposure data and genome-wide association studies data on KOA to evaluate the impact of antihypertensive drugs on KOA. We observed a significant association between decreased expression of the *SLC12A2* target gene and a reduced risk of KOA (odds ratio: 0.915, 95% confidence interval: 0.869–0.964, *P* < .001). In this study, we find that *SLC12A2* inhibitors can have a beneficial effect on KOA, and that the *SLC12A2* gene may be a potential therapeutic target for KOA. These findings suggest that when treating patients with both hypertension and KOA, clinicians may consider prioritizing the use of *SLC12A2* inhibitors.

## 1. Introduction

Knee osteoarthritis (KOA) is a chronic musculoskeletal disease and common joint disease of older adults worldwide.^[1,2]^ Common clinical presentations of KOA include knee pain, swelling, stiffness, and limited joint function.^[3]^ It can have a negative impact on both physical and mental well-being, with a high likelihood of resulting in disability.^[2,4,5]^ However, current treatments are limited to improving joint function and relieving pain without the ability to cure the disease completely. The prevalence and incidence of KOA vary across different research attributed to variations in the definitions of KOA, differences in study populations, and other contributing factors. It is noteworthy that numerous studies have demonstrated a consistent increase in the prevalence of KOA year after year.^[6–8]^

In cardiovascular diseases, hypertension plays a pivotal role. Globally, the prevalence of hypertension stands at approximately 30%, with projections indicating an alarming rise to 60% by 2025.^[9,10]^ While the primary diagnostic criteria for hypertension revolve around systolic blood pressure (SBP) and diastolic blood pressure (DBP), recent studies have underscored that SBP surpasses DBP in its predictive capacity for hypertension-related risks.^[11–13]^ The main approaches for managing blood pressure (BP) involve lifestyle modifications and the utilization of antihypertensive medications. Commonly prescribed antihypertensive drugs include angiotensin-converting enzyme inhibitors (ACEIs), angiotensin receptor blockers (ARBs), β-receptor blockers (BBs), calcium channel blockers (CCBs), and diuretics.^[12,14,15]^ However, due to the fact that BBs have a lower blood pressure-lowering effect and safety profile than other commonly used antihypertensive drugs, BBs are considered not to be the first-line choice.^[16–18]^ Despite the significant efficacy of current treatment regimens in reducing patients’ blood pressure, some individuals still have difficulty controlling their blood pressure within the ideal range, which may be related to patient adherence and drug selection.^[18]^

Hypertension and KOA are prevalent chronic conditions, and recent studies have revealed a strong association between the two. A study has demonstrated that hypertension may serve as a risk factor for OA and is positively correlated with the severity of OA-related pain.^[19]^ However, another study has indicated that KOA can increase the risk of hypertension, potentially due to pathological changes in the extracellular matrix following KOA onset.^[20]^ In recent years, several researchers have conducted experimental studies investigating the impact of antihypertensive drugs on osteoarthritis (OA). Through cellular and animal experiments, they have discovered that certain antihypertensive medications exhibit potential for improving OA.^[21–23]^ However, it is important to note that thus far, there has been a lack of large-scale clinical studies to substantiate this perspective. The present study employed drug target MR analysis to find the association between antihypertensive drugs and KOA, aiming to offer guidance and assistance for the concomitant therapy of patients with both hypertension and KOA.

Mendelian randomization (MR) is often described as a naturally occurring randomized controlled trial that utilizes the inherent random distribution between genotype and phenotype to mitigate confounding factors in human trials to some extent.^[24]^ Drug target MR simulates potential drug effects by employing genetic instruments located near or within the target gene, enabling the investigation of whether the genetic susceptibility of a designated drug target influences disease risk without being influenced by potential confounders.^[25]^ This study aims to explore the causal relationship between 4 primary antihypertensive drugs and KOA using recently published genome-wide association study (GWAS) statistics.

## 2. Materials and methods

### 2.1. Selection of genetic instruments

The objective of this study is to explore the causal association between 4 primary antihypertensive agents (ACEIs, ARBs, CCBs, and diuretics) and KOA, with SBP as a medium. The data utilized in this study were publicly accessible, with summary statistics on BP-related GWAS available from the IEU Open GWAS Project database (ieu-b-38).^[26]^ Instrumental variables (IVs) targeting gene loci associated with SBP reduction were employed to simulate the effects of the 4 antihypertensive agents. The selection process for IVs for different antihypertensive agents followed these steps: Firstly, 25 target genes for ACEIs, ARBs, CCBs, and diuretics were obtained from the Drugbank online database (see Table S1, Supplemental Digital Content, https://links.lww.com/MD/Q389). Secondly, chromosomal locations of these target genes were acquired from the NCBI gene database. Finally, a tool was introduced to substitute exposure to antihypertensive agents, selecting SBP-related SNPs at a genome-wide significance level (*P* < 5 × 10^−8^) within a 100 kb window surrounding each agent’s target genes. We allowed low-weak linkage disequilibrium (LD threshold *R*^2^ < 0.3) between SNPs used as IVs in order to maximize instrument validity. On this basis, we further calculated the *F*-statistic for each IV and used a threshold of *F* > 10 to exclude any bias caused by weak IVs.

Statistical data for KOA were obtained from the IEU Open GWAS Project database(ebi-a-GCST007090). This GWAS summary statistics included 403,124 samples (24,955 cases and 378,169 controls) and 29,999,696 SNPs. To ensure the accuracy of the MR analysis, we also selected another GWAS dataset from the UK Biobank for replication(ebi-a-GCST005813). This GWAS dataset includes 22,347 samples (4462 cases and 17,885 controls) and 157,086,90 SNPs.

### 2.2. Data analysis

We initially integrated the exposure-related drug-targeted IVs with the outcome dataset and then employed online website PhenoScanner(http://www.phenoscanner.medschl.cam.ac.uk/)^[27]^ and MR-PRESSO to Remove confounding factors. Subsequently, we conducted MR analysis using 3 statistical methods: inverse-variance weighted (IVW), MR Egger, and weighted median; IVW was considered the primary test method. Cochran q-tests were then utilized to assess heterogeneity presence, where *P* > .05 indicated its absence. To evaluate the potential presence of horizontal pleiotropy, we conducted the MR-Egger test, which would indicate the presence of horizontal pleiotropy if *P* < .05. Finally, a leave-one-out sensitivity analysis was performed to ensure the stability of our MR results by examining whether the removal of any IV significantly affected the results. All results were reported as odds ratio (OR) with a corresponding 95% confidence interval (CI). Statistical significance was defined at *P* < .05 for all analyses conducted in this Mendelian randomization study. The R version 4.2.3 was utilized in this study, along with the R packages “TwoSampleMR,” “MRPRESSO.”

## 3. Result

### 3.1. Identification of IVs

**Figure 1.**
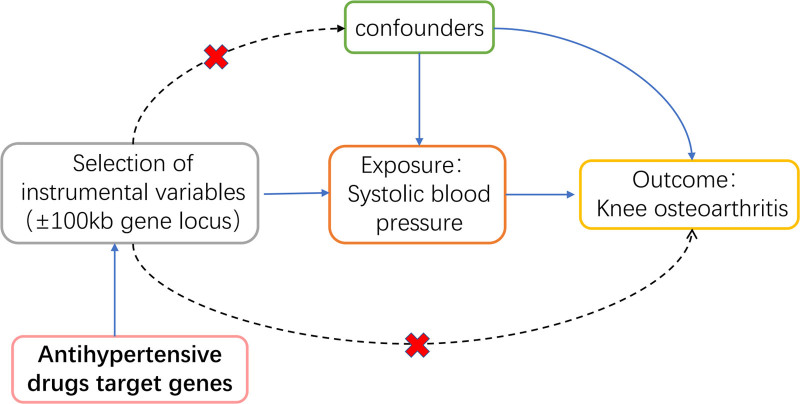
The design strategy of this drug target Mendelian randomization study. Three conditions must be satisfied: instrumental variables show no association with confounders (marked with a dashed line); instrumental variables are associated with the exposure factor (marked with a solid line); and instrumental variables have no direct association with the outcome (marked with a dashed line).

The design strategy of this study is illustrated in Figure 1. We identified the target genes of ACEIs, ARBs, CCBs, and diuretics using Drugbank (https://go.drugbank.com/) and determined the chromosomal locations of these target genes using NCBI (https://www.ncbi.nlm.nih.gov/) (see Table S2, Supplemental Digital Content, https://links.lww.com/MD/Q389). Subsequently, we located blood pressure-related SNPs in the vicinity of the drug target genes for screening IVs associated with the drugs. Furthermore, we summarized the exposure and outcome data and employed MR-PRESSO to identify outliers and eliminate biased SNPs. Additionally, it was stipulated that if fewer than 5 SNPs were available for MR analysis, the corresponding exposure would be excluded. The IVs eligible for MR analysis are summarized in Supplementary Table S3 and Supplementary Table S4 Supplemental Digital Content, https://links.lww.com/MD/Q389.

### 3.2. Causal effects of SBP on knee osteoarthritis mediated by antihypertensive drugs

**Figure 2.**
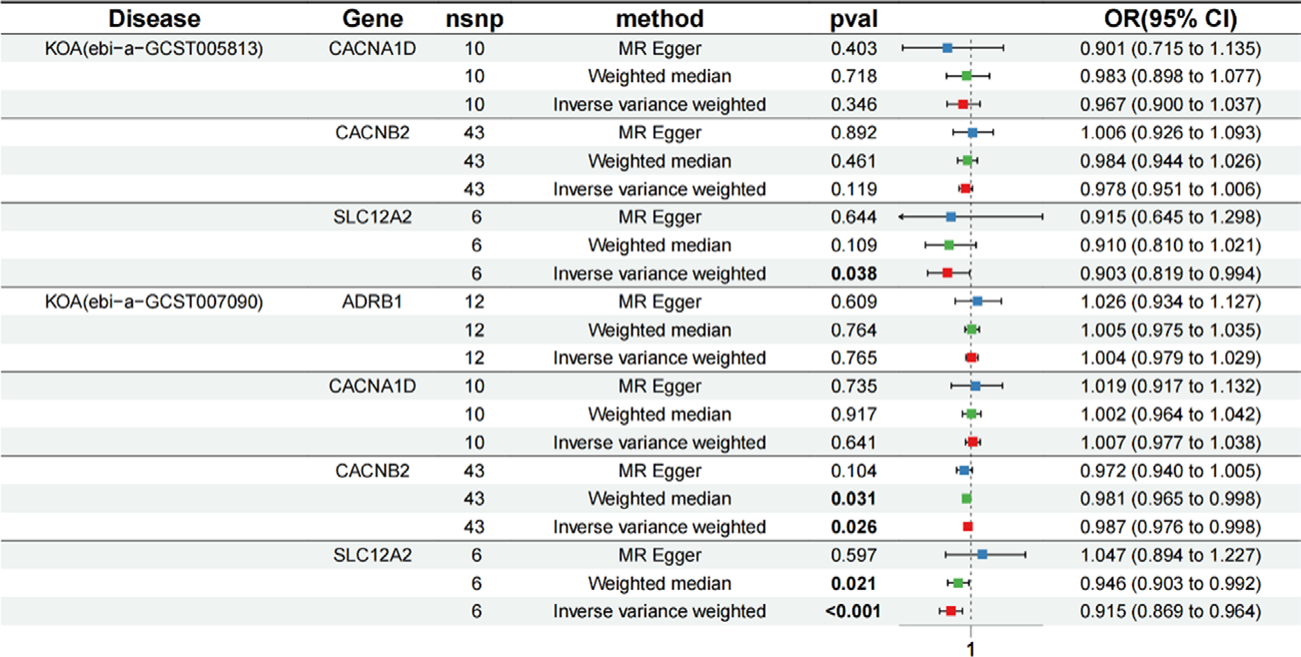
The causal relationship between antihypertensive drugs and KOA was examined using 3 regression models. ADRB1 = adrenergic receptor beta 1, CACNA1D = calcium voltage-gated channel subunit alpha1 D, CACNB2 = calcium voltage-gated channel auxiliary subunit beta 2, CI = confidence interval, KOA = knee osteoarthritis, OR = odds ratio, SLC12A2 = solute carrier family 12 member 2.

The causal relationship between antihypertensive drugs and KOA was examined using 3 regression models, as illustrated in Figure 2. The results of the MR analysis indicated no evidence of a causal relationship between CCBs’ target gene *CACNA1D* and the risk of KOA, with all *P*-values obtained from the 3 tests exceeding 0.05(IVW: *P* = .641). Subsequently, we investigated another target gene of CCBs, namely *CACNB2*, and found that the IVW test revealed an OR of 0.987, accompanied by a 95% CI ranging from 0.976 to 0.998, with *P* = .026. The result suggests that *CACNB2* may mitigate KOA risk through SBP regulation. Similar outcomes were observed in the other 2 tests. However, only the Weighted median test demonstrated a significant association (*P* = .031). The target gene of diuretics, *SLC12A2*, was also found to exhibit a significant causal association with KOA. The IVW test yielded an OR of 0.915, with a 95%CI ranging from 0.869 to 0.964 and *P* < .001. Similarly, the weighted median test demonstrated an OR of 0.946, with a 95%CI from 0.903 to 0.992 and *P* = .021. These findings suggest that *SLC12A2*, a target gene, can potentially reduce KOA risk via SBP regulation.

To validate the reliability of the MR findings, we conducted an MR analysis on exposure and another KOA outcome. The IVW results demonstrated a significant causal association between *SLC12A2* and KOA, suggesting that this target could mitigate the risk of KOA by regulating blood pressure (IVW: OR = 0.903, 95% CI = 0.819–0.994, *P* = .038). However, regrettably, we were unable to replicate the noteworthy correlation between the *CACNB2* gene and KOA (*P* = .119). Based on our validation process, we can tentatively conclude that *SLC12A2*, as a diuretic target, has the potential to reduce the risk of KOA through SBP regulation.

### 3.3. Sensitivity analysis

**Figure 3.**
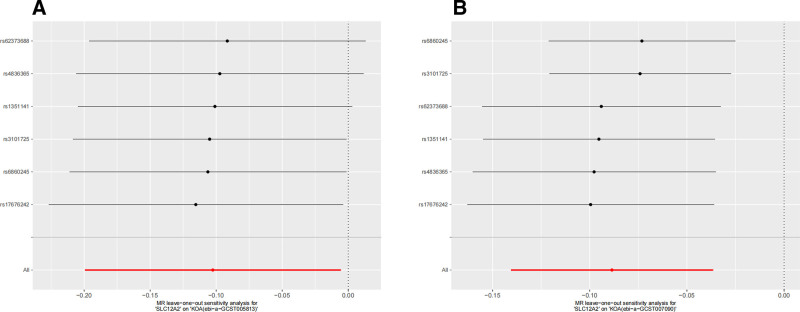
Sensitivity analysis of SLC12A2 on KOA (The leave-one-out tests). (A) “SLC12A2” on “KOA” (ebi-a-GCST007090); (B) “SLC12A2” on “KOA” (ebi-a-GCST005813). KOA = knee osteoarthritis, SLC12A2 = solute carrier family 12 member 2.

After conducting the MR analysis, we employed the Cochran Q and MR Egger methods to examine the heterogeneity and horizontal pleiotropy. If both tests yielded *P* > .05, it indicated that there was no heterogeneity or horizontal pleiotropy in the analysis results, allowing for further investigation. The outcomes revealed that all test methods produced *P* > .05 (Cochran Q: *P* = .089; MR Egger: *P* = .157), suggesting an absence of heterogeneity and horizontal pleiotropy in the IVs included in the aforementioned study results. Consequently, we proceeded with a leave-one-out test to assess whether removing each IV individually affected the significance of the MR analysis results. The leave-one-out tests demonstrated stability in the MR analysis results, thereby providing additional confirmation regarding the reliability of the association between the *SLC12A2* gene and KOA (see Fig. 3).

## 4. Discussion

To investigate whether antihypertensive drugs would affect KOA in patients with hypertension and KOA, we integrated relevant GWAS data (consisting of individuals of European ancestry), information on the targets of antihypertensive drugs, and data on the expression of target genes. Through initial MR analysis, we found that the expression of the target gene of CCBs, *CACNB2*, and the target gene of diuretics, *SLC12A2*, were both associated with a lower risk of developing KOA. Unfortunately, we were only able to replicate the significant association between *SLC12A2* and KOA in the replication dataset. We hypothesized that this might be due to the fact that contemporary Europeans originate from multiple genetically differentiated subpopulations. Still, to ensure the generalizability of our findings, we chose to exclude the unreplicated *CACNB2* gene. We then conducted a sensitivity analysis on the *SLC12A2* gene and KOA, and the results showed that the MR analysis was stable. Based on this analysis, we conclude that the target gene *SLC12A2* is a potential therapeutic target for KOA.

The precise mechanism of KOA remains incompletely understood. Still, it is widely believed among scholars that it is associated with aging, environmental factors, biomechanical influences, and biochemical changes. The pathogenesis of this disease is considered to be multifactorial, with initial stages primarily characterized by the release of inflammatory mediators, infiltration and erosion of cartilage tissue leading to chondrocyte death, and alterations in cartilage matrix composition. Ultimately, these processes culminate in the destruction of cartilage structure.^[28]^ Furthermore, the damage to subchondral bone also plays a significant role in the pathological progression of KOA. During KOA development, various pathological conditions arise, including altered blood flow supply, imbalanced bone remodeling, bone marrow edema, and increased osteoclast activity within the subchondral bone. These factors contribute to structural changes within the bone tissue.^[29,30]^ Hypertension has been implicated as a potential inducer or exacerbator for KOA. Currently, the discussion on the role of hypertension in KOA is mainly focused on the blood flow changes in joint tissue. Hypertension will cause pathological changes in the microvasculature nourishing the subchondral bone, such as vascular narrowing and thrombosis, which will lead to metabolic abnormalities in the tissues supplied by the vessels, such as nearby bone tissue and articular cartilage, and cause or aggravate joint tissue damage.^[31,32]^ In addition to affecting joint tissue metabolism, hypertension also increases intra-bone pressure, further aggravating bone marrow edema, ultimately causing damage to the calcified cartilage layer while accelerating bone–cartilage crosstalk, thereby disrupting their structural integrity.^[32–34]^

Diuretics are commonly prescribed medications for hypertension, and they can be categorized into loop diuretics, thiazide diuretics, and potassium-sparing diuretics, among others. The gene *SLC12A2* we obtained in this study is a target gene for loop diuretics, and bumetanide and torasemide are closely related to this gene. *SLC12A2* encodes the Na^+^-K^+^-2Cl^-^ cotransporter-1 (NKCC1), which exerts physiological and pathological effects through NKCC1. Unlike NKCC2, which is predominantly expressed in the kidneys, NKCC1 is believed to have ubiquitous distribution.^[35]^ However, unfortunately, there are currently no large-scale clinical studies investigating the role of NKCC1 in OA. Thus, we can only speculate on its potential mechanisms of action.

The beneficial effects of NKCC1 inhibitors on KOA may be related to changes in blood flow to subchondral bone, anti-edema, and inhibition of pain perception after drug application. Multiple studies have shown that NKCC1 inhibitors can lower systemic blood pressure by reducing the tone of the portal vein and resistance vessels.^[36–38]^ To verify that the decrease in blood pressure was not due to the diuretic function of the drug, they also clamped the renal artery in the experiment and found that bumetanide still had a significant hypotensive effect.^[36]^ The vasodilating effect of NKCC1 inhibitors on resistance vessels may have a beneficial impact on blood flow to subchondral bone. Relaxing the contracted small arteries and micro-arteries facilitates the exchange of fluids, gases, and nutrients in the subchondral tissue, thereby mitigating bone marrow edema and cartilage damage.

In the KOA state, the tissue releases large amounts of inflammatory factors, and IL-6 will stimulate the activity of the fibroblast-like synoviocyte surface NKCC1, inducing cell swelling and leading to synovial tissue edema.^[39,40]^ Multiple studies have found that inhibiting NKCC1 can alleviate spinal and cerebral edema, but there is no direct evidence proving that NKCC1 inhibitors can relieve joint edema in inflammatory states.^[41,42]^ An experimental study discovered that intracerebroventricular injection of histamine or glutamate into the ventral tuberomammillary nucleus exacerbates arthritis-induced joint edema, while intracerebroventricular injection of bumetanide could inhibit its enhancement.^[43]^ NKCC1 has excellent potential in anti-edema; based on existing research, we hypothesize that NKCC1 inhibitors may reduce cellular accumulation of Na^+^ and Cl^-^ by inhibiting the activity of NKCC1 protein on the surface of fibroblast-like synoviocyte, thereby alleviating tissue edema. In addition to these pathways, reducing central and peripheral tissues’ sensitivity to pain and interfering with 1α,25-dihydroxyvitamin D3 pathway-induced osteoclastogenesis may also be ways through which NKCC1 inhibitors participate in OA’s pathogenesis and progression.^[44–46]^

CACNB2 is a therapeutic target of CCBs. Studies have indicated that nifedipine, a CACNB2-related drug, exerts a positive effect on cartilage repair under inflammatory conditions.^[22,47]^ Unfortunately, we failed to replicate the association between CACNB2 and KOA in the validation dataset, which may be attributed to several factors: 1. Contemporary European populations originate from multiple genetically differentiated subpopulations, and the data sources of the 2 cohorts are not entirely identical. This could lead to differences in results due to variations in genetics, diet, and other aspects among the study populations; 2. The sample sizes of the 2 databases differ, with the sample size of the validation cohort being much smaller than that of the discovery cohort. This may result in insufficient statistical power in the validation cohort to detect the true but weak effect identified in the discovery cohort. These factors may cause variations in the strength of the CACNB2 gene-phenotype association across cohorts. However, we believe this does not directly negate the potential research value of CACNB2. In the future, the association could be reanalyzed using larger-scale and more homogeneous cohorts, or trans-ethnic Mendelian randomization analysis could be conducted using cohort data from other populations to verify whether the association of CACNB2 is conserved across different ethnicities.

Ethnicity is a factor that cannot be ignored in the process of drug development and application. Multiple studies have shown that significant efficacy differences of the same drug exist among different ethnic groups. Such differences may be related to genetic variations between ethnic groups, or influenced by external factors such as diet and environment. The target population of this study is European populations. Considering the efficacy differences caused by ethnicity, SLC12A2 may currently only be suitable as a therapeutic target for KOA in individuals of European ancestry. Clinically, it may be advisable to prioritize the use of NKCC1 inhibitors such as Bumetanide and Torasemide in high-risk KOA patients with hypertension or in those already diagnosed with KOA, and to conduct relevant clinical trials for validation. It is important to note that, in the absence of additional evidence, caution should be exercised when extending these findings to populations of other ancestries.

The study has several advantages. Firstly, we employed MR analysis to effectively control for confounding factors and utilized multiple detection methods to ensure the stability of our analysis results. Secondly, we identified IVs of drug targets by screening SNPs in the vicinity of the target genes that were associated with SBP, which resulted in IVs that were both related to systolic blood pressure and drug targets, making the study results more reliable. Thirdly, we replicated our MR results on the validation dataset, which further ensures the reliability of our MR analysis. However, our study has certain limitations as well. Firstly, the study subjects consisted mainly of European populations. Therefore, generalizing our research findings to other populations may be challenging. Secondly, despite extensive efforts to minimize confounding factors through MR analysis, residual and unmeasured confounders might still exist.

## 5. Conclusion

In summary, our MR analysis of 4 classes of antihypertensive drugs and KOA revealed a positive correlation between *SLC12A2* expression and the risk of KOA. Therefore, we suggest that selecting appropriate NKCC1 inhibitors, such as bumetanide and torasemide, for hypertensive patients with KOA may lead to better therapeutic outcomes. And the *SLC12A2* gene may be a potential therapeutic target for KOA.

## Acknowledgments

The authors thank the IEU open GWAS project and the UK Biobank.

## Author contributions

**Conceptualization:** Zhixin Li, Yong Ma.

**Data curation:** Mengmin Liu, Pengcheng Tu.

**Formal analysis:** Mengmin Liu, Pengcheng Tu.

**Funding acquisition:** Yong Ma.

**Methodology:** Yafeng Zhang, Yuhao Si.

**Project administration:** Mengmin Liu, Yang Guo.

**Software:** Zhixin Li, Yuhao Si, Yafeng Zhang.

**Validation:** Yang Guo, Yong Ma.

**Writing – original draft:** Zhixin Li.

**Writing – review & editing:** Yang Guo.

## Supplementary Material


