# Supplementary material for: The causal relationship between antihypertensive drugs and knee osteoarthritis: A drug target Mendelian randomization study
Source: Medicine (Baltimore). 2025 Oct 17;104(42):e45328. doi: 10.1097/MD.0000000000045328 (PMC12537083; doi:10.1097/MD.0000000000045328)
Supplement: Supplementary file 1 [file medi-104-e45328-s001.docx]

Supplementary Table S1. Classes of anti-hypertensive drugs and target genes

| **Drug class** | **Anti-hypertensive drug** | **DrugBank accession number** | **Target gene** |
| --- | --- | --- | --- |
| ACE inhibitor | Enalaprilat | DB09477 | ACE |
| ACE inhibitor | Cilazapril | DB01340 | ACE |
| ACE inhibitor | Perindopril | DB00790 | ACE |
| ACE inhibitor | Trandolapril | DB00519 | ACE |
| ACE inhibitor | Zofenopril | DB13166 | ACE |
| ACE inhibitor | Moexipril | DB00691 | ACE |
| ACE inhibitor | Quinapril | DB00881 | ACE |
| ACE inhibitor | Ramipril | DB00178 | ACE |
| ACE inhibitor | Spirapril | DB01348 | ACE |
| ACE inhibitor | Captopril | DB01197 | ACE |
| ACE inhibitor | Benazepril | DB00542 | ACE |
| ACE inhibitor | Fosinopril | DB00492 | ACE |
| ACE inhibitor | Enalapril | DB00584 | ACE |
| Angiotensin II receptor antagonist | Sparsentan | DB12548 | AGTR2 |
| Angiotensin II receptor antagonist | Eprosartan | DB00876 | AGTR1 |
| Angiotensin II receptor antagonist | Telmisartan | DB00966 | AGTR1 |
| Angiotensin II receptor antagonist | Irbesartan | DB01029 | AGTR1 |
| Angiotensin II receptor antagonist | Candesartan cilexetil | DB00796 | AGTR1 |
| Angiotensin II receptor antagonist | Azilsartan medoxomil | DB08822 | AGTR1 |
| Calcium channel blocker | Nilvadipine | DB06712 | CACNA1C, CACNA2D1, CACNB2 |
| Calcium channel blocker | Bepridil | DB01244 | CACNA1A, CACNA1H, CACNA2D2 |
| Calcium channel blocker | Nisoldipine | DB00401 | CACNA1C, CACNA2D1, CACNB2, CACNA1D, CACNA1S |
| Calcium channel blocker | Nifedipine | DB01115 | CACNA1C, CACNA1D, CACNB2 |
| Calcium channel blocker | Nimodipine | DB00393 | CACNA1C, CACNA1D, CACNA1F, CACNA1S, CACNB1, CACNB2, CACNB3, CACNB4 |
| Calcium channel blocker | Verapamil | DB00661 | CACNA1C |
| Calcium channel blocker | Felodipine | DB01023 | CACNA1C, CACNA2D1, CACNB2, CACNA1D, CACNA1S |
| Calcium channel blocker | Lercanidipine | DB00528 | CACNG1 |
| Calcium channel blocker | Nitrendipine | DB01054 | CACNA1C, CACNA2D1, CACNB2, CACNA1D, CACNA1S |
| Calcium channel blocker | Nicardipine | DB00622 | CACNA1C, CACNB2, CACNA2D1, CACNA1D |
| Calcium channel blocker | Diltiazem | DB00343 | CACNA1C, CACNG1 |
| Diuretics | Trichlormethiazide | DB01021 | SLC12A3 |
| Diuretics | Chlorothiazide | DB00880 | SLC12A3 |
| Diuretics | Quinethazone | DB01325 | SLC12A1, SLC12A2, SLC12A3 |
| Diuretics | Hydrochlorothiazide | DB00999 | SLC12A3 |
| Diuretics | Hydroflumethiazide | DB00774 | SLC12A1 |
| Diuretics | Bumetanide | DB00887 | SLC12A1, SLC12A2, SLC12A4, SLC12A5 |
| Diuretics | Torasemide | DB00214 | SLC12A1, SLC12A2 |
| Diuretics | Triamterene | DB00384 | SCNN1A, SCNN1B, SCNN1G |
| Diuretics | Amiloride | DB00594 | SCNN1A, SCNN1B, SCNN1D, SCNN1G |

Supplementary Table S2. Regions of target genes identified by the NCBI Gene database

| **Drug class** | **Target gene** | **Chromosome** | **Position (GRCh37/hg19)** |
| --- | --- | --- | --- |
| ACE inhibitor | ACE | 17 | 61554422-61575734 |
| Angiotensin II receptor antagonist | AGTR1 | 3 | 148415690-148460790 |
|  | AGTR2 | X | 115301997-115306227 |
| Calcium channel blocker | CACNA1A | 19 | 13317256-13617293 |
|  | CACNA1C | 12 | 2162153-2807116 |
|  | CACNA1D | 3 | 53528638-53847760 |
|  | CACNA1F | X | 49061523-49089802 |
|  | CACNA1H | 16 | 1203106-1271768 |
|  | CACNA1S | 1 | 201008640-201081554 |
|  | CACNA2D1 | 7 | 81575760-82073272 |
|  | CACNA2D2 | 3 | 50400044-50541675 |
|  | CACNB1 | 17 | 37329706-37353922 |
|  | CACNB2 | 10 | 18429353-18832486 |
|  | CACNB3 | 12 | 49208263-49222724 |
|  | CACNB4 | 2 | 152689285-152955681 |
|  | CACNG1 | 17 | 65040670-65052913 |
| Diuretics | SLC12A1 | 15 | 48498499-48596275 |
|  | SLC12A2 | 5 | 127419458-127525369 |
|  | SLC12A3 | 16 | 56899119-56949762 |
|  | SLC12A4 | 16 | 67977377-68002597 |
|  | SLC12A5 | 20 | 44650325-44688789 |
|  | SCNN1A | 12 | 6456013-6486525 |
|  | SCNN1B | 16 | 23313623-23392615 |
|  | SCNN1D | 1 | 1215816-1227405 |
|  | SCNN1G | 16 | 23194066-23228204 |

Supplementary Table S3. Details of the IVs used for MR analysis [causal effect of SBP on KOA (ebi-a-GCST007090)]

| **Gene** | **SNP** | **Chr** | **Pos** | **EA** | **OA** | **Exposure** | | | | **Outcome** | | | |
| --- | --- | --- | --- | --- | --- | --- | --- | --- | --- | --- | --- | --- | --- |
|  |  |  |  |  |  | **Beta** | **SE** | ***P*-value** | **EAF** | **Beta** | **SE** | ***P*-value** | **EAF** |
| ADRB1 | rs10885531 | 10 | 115814392 | T | C | 0.2999 | 0.0302 | 3.63E-23 | 0.5009 | 2.00E-04 | 0.0093 | 0.983 | 0.4968 |
|  | rs11196553 | 10 | 115710997 | T | C | 0.6252 | 0.074 | 2.89E-17 | 0.045 | -0.0041 | 0.0227 | 0.8556 | 0.0437 |
|  | rs11196597 | 10 | 115788094 | A | G | 0.2858 | 0.0458 | 4.23E-10 | 0.133 | 0.0033 | 0.0138 | 0.8109 | 0.1289 |
|  | rs11196625 | 10 | 115843990 | A | G | 0.2274 | 0.0395 | 8.29E-09 | 0.1928 | -0.0131 | 0.0117 | 0.2662999 | 0.1987 |
|  | rs143854972 | 10 | 115843445 | A | G | 0.4306 | 0.0664 | 8.89E-11 | 0.0578 | -0.0046 | 0.02 | 0.8171 | 0.0581 |
|  | rs17875473 | 10 | 115800294 | T | C | 0.3283 | 0.0552 | 2.66E-09 | 0.0871 | -0.0109 | 0.0169 | 0.5203002 | 0.0821 |
|  | rs180898 | 10 | 115765397 | C | A | -0.5177 | 0.065 | 1.65E-15 | 0.0661 | -0.0495 | 0.0195 | 0.0111099 | 0.0636 |
|  | rs460718 | 10 | 115721364 | G | A | 0.2764 | 0.0324 | 1.36E-17 | 0.6734 | 0.0138 | 0.0099 | 0.1653001 | 0.6724 |
|  | rs68122733 | 10 | 115831533 | G | A | -0.3343 | 0.0406 | 1.69E-16 | 0.1718 | 0.0188 | 0.0123 | 0.1271001 | 0.1728 |
|  | rs740746 | 10 | 115792787 | A | G | 0.4557 | 0.0342 | 1.42E-40 | 0.7318 | 0.0037 | 0.0105 | 0.7224004 | 0.7347 |
|  | rs79850079 | 10 | 115790006 | A | G | -0.5804 | 0.0905 | 1.45E-10 | 0.0317 | 0.0422 | 0.0269 | 0.1160999 | 0.0306 |
|  | rs855715 | 10 | 115823524 | T | G | -0.4394 | 0.0482 | 7.88E-20 | 0.1202 | -0.0125 | 0.0145 | 0.3869002 | 0.1207 |
| CACNA1D | rs113210396 | 3 | 53612327 | T | G | -0.4338 | 0.077 | 1.76E-08 | 0.0451 | 0.0053 | 0.0223 | 0.8135001 | 0.0455 |
|  | rs114718455 | 3 | 53464055 | G | A | 0.5102 | 0.0905 | 1.72E-08 | 0.0336 | -0.028 | 0.0251 | 0.2640998 | 0.0351 |
|  | rs114987861 | 3 | 53605712 | A | G | 0.5289 | 0.0958 | 3.36E-08 | 0.0284 | -0.0022 | 0.0292 | 0.9386001 | 0.0268 |
|  | rs2633731 | 3 | 53738424 | C | T | 0.1963 | 0.0309 | 2.21E-10 | 0.6038 | -0.0062 | 0.0095 | 0.5144996 | 0.5989 |
|  | rs312487 | 3 | 53545622 | C | T | -0.2194 | 0.0307 | 9.65E-13 | 0.5217 | -0.0024 | 0.0094 | 0.7975 | 0.5239 |
|  | rs3774472 | 3 | 53638200 | G | A | 0.1747 | 0.0303 | 8.21E-09 | 0.4943 | -0.0043 | 0.0094 | 0.6454001 | 0.493 |
|  | rs3821843 | 3 | 53558012 | A | G | 0.3373 | 0.0335 | 6.56E-24 | 0.6808 | 0.0127 | 0.0101 | 0.2069998 | 0.6789 |
|  | rs62250937 | 3 | 53870318 | C | T | -0.3238 | 0.056 | 7.28E-09 | 0.0893 | -0.0219 | 0.0167 | 0.1888 | 0.0887 |
|  | rs7340705 | 3 | 53734443 | C | T | 0.2425 | 0.0322 | 4.87E-14 | 0.3268 | -0.0131 | 0.01 | 0.1894999 | 0.3225 |
|  | rs9311502 | 3 | 53560321 | C | T | 0.2463 | 0.0355 | 3.87E-12 | 0.2391 | 0.0218 | 0.0109 | 0.0464205 | 0.2355 |
| CACNB2 | rs10764319 | 10 | 18428415 | T | C | 0.2693 | 0.0329 | 2.54E-16 | 0.3044 | -0.0103 | 0.0102 | 0.3151998 | 0.2949 |
|  | rs10828399 | 10 | 18553968 | A | G | -0.1947 | 0.0302 | 1.10E-10 | 0.5218 | 0.0131 | 0.0093 | 0.1581 | 0.5125 |
|  | rs10828452 | 10 | 18592450 | T | A | -0.3046 | 0.0388 | 4.20E-15 | 0.207 | 0.0155 | 0.0116 | 0.1824001 | 0.2085 |
|  | rs10828542 | 10 | 18627285 | G | A | -0.1817 | 0.0311 | 5.18E-09 | 0.3863 | 0.0035 | 0.0096 | 0.7173994 | 0.3866 |
|  | rs10828749 | 10 | 18756881 | A | G | -0.3658 | 0.0309 | 2.27E-32 | 0.412 | -0.0059 | 0.0095 | 0.5327004 | 0.4108 |
|  | rs10828784 | 10 | 18788273 | G | C | -0.2021 | 0.0345 | 4.49E-09 | 0.3367 | -0.0123 | 0.0102 | 0.2292002 | 0.3414 |
|  | rs11012811 | 10 | 18438456 | T | G | 0.3095 | 0.0326 | 2.31E-21 | 0.31 | -0.0114 | 0.0101 | 0.2582998 | 0.3047 |
|  | rs11013910 | 10 | 18663370 | A | G | 0.2672 | 0.0449 | 2.70E-09 | 0.1306 | -0.001 | 0.0137 | 0.9414 | 0.1335 |
|  | rs11013938 | 10 | 18669271 | C | G | -0.3265 | 0.035 | 1.17E-20 | 0.2554 | 0.0335 | 0.0107 | 0.001679 | 0.2588 |
|  | rs11014021 | 10 | 18682233 | A | T | -0.3245 | 0.0307 | 4.29E-26 | 0.4182 | 0.0075 | 0.0095 | 0.4278002 | 0.4149 |
|  | rs112133583 | 10 | 18695681 | T | C | -0.5546 | 0.0973 | 1.18E-08 | 0.0299 | 0.0102 | 0.0272 | 0.7068007 | 0.0315 |
|  | rs112701401 | 10 | 18644811 | G | C | -0.5026 | 0.0921 | 4.91E-08 | 0.0305 | -7.00E-04 | 0.0273 | 0.9804 | 0.0308 |
|  | rs116936375 | 10 | 18737135 | A | G | -0.5739 | 0.081 | 1.40E-12 | 0.0405 | 0.0208 | 0.0245 | 0.3948999 | 0.0375 |
|  | rs12258967 | 10 | 18727959 | G | C | -0.6327 | 0.0337 | 1.08E-78 | 0.2953 | 0.0155 | 0.0101 | 0.1266001 | 0.2986 |
|  | rs12416030 | 10 | 18789075 | C | T | 0.2088 | 0.0381 | 4.32E-08 | 0.2031 | -0.0242 | 0.0117 | 0.03843 | 0.2025 |
|  | rs12416052 | 10 | 18789267 | C | T | -0.1987 | 0.0311 | 1.59E-10 | 0.4053 | -0.0067 | 0.0095 | 0.4812004 | 0.4088 |
|  | rs12765240 | 10 | 18438372 | T | C | 0.396 | 0.052 | 2.52E-14 | 0.0932 | 0.0082 | 0.0159 | 0.6085006 | 0.0941 |
|  | rs12778700 | 10 | 18385490 | C | T | -0.1972 | 0.033 | 2.24E-09 | 0.3323 | -0.0203 | 0.0099 | 0.03992 | 0.3411 |
|  | rs1539680 | 10 | 18502889 | C | G | -0.3259 | 0.0375 | 3.37E-18 | 0.7929 | -0.0159 | 0.0115 | 0.1663999 | 0.797 |
|  | rs16916922 | 10 | 18467744 | T | A | 0.3662 | 0.0433 | 2.86E-17 | 0.1415 | -1.00E-04 | 0.0134 | 0.9914 | 0.1397 |
|  | rs1757213 | 10 | 18537594 | G | A | -0.3084 | 0.0507 | 1.15E-09 | 0.888 | -0.012 | 0.0145 | 0.4101002 | 0.8812 |
|  | rs1757225 | 10 | 18516925 | G | A | -0.2453 | 0.0325 | 4.29E-14 | 0.6855 | -0.006 | 0.01 | 0.5471004 | 0.6903 |
|  | rs17604757 | 10 | 18442940 | G | A | 0.5022 | 0.0606 | 1.12E-16 | 0.0675 | -0.0254 | 0.0189 | 0.1785998 | 0.0648 |
|  | rs17610275 | 10 | 18621630 | G | T | -0.3868 | 0.0613 | 2.87E-10 | 0.0734 | 0.0061 | 0.0184 | 0.7379994 | 0.0715 |
|  | rs1888693 | 10 | 18440444 | A | G | 0.3858 | 0.0317 | 4.69E-34 | 0.3449 | -7.00E-04 | 0.0098 | 0.9458 | 0.343 |
|  | rs1891392 | 10 | 18336421 | C | T | -0.2276 | 0.0334 | 9.02E-12 | 0.7129 | 0.0173 | 0.0102 | 0.0895097 | 0.7086 |
|  | rs1998822 | 10 | 18755664 | G | A | 0.1958 | 0.0343 | 1.15E-08 | 0.2766 | -0.015 | 0.0105 | 0.1504998 | 0.2795 |
|  | rs4748444 | 10 | 18494482 | C | T | -0.1939 | 0.0327 | 3.13E-09 | 0.3363 | -0.0042 | 0.0098 | 0.6687996 | 0.3394 |
|  | rs4748472 | 10 | 18776197 | T | C | 0.3161 | 0.0319 | 4.04E-23 | 0.6558 | -0.0062 | 0.0098 | 0.5292002 | 0.6544 |
|  | rs61278674 | 10 | 18481737 | G | A | 0.3298 | 0.054 | 1.03E-09 | 0.0938 | -0.0095 | 0.016 | 0.5524996 | 0.0977 |
|  | rs6482184 | 10 | 18403118 | C | T | 0.1949 | 0.0316 | 6.65E-10 | 0.3795 | 0.0049 | 0.0096 | 0.6138002 | 0.3728 |
|  | rs67214975 | 10 | 18727251 | A | C | -0.4144 | 0.0307 | 1.42E-41 | 0.4563 | 0.0124 | 0.0093 | 0.1831998 | 0.46 |
|  | rs7074171 | 10 | 18676897 | G | A | 0.3061 | 0.0314 | 1.76E-22 | 0.5795 | 3.00E-04 | 0.0096 | 0.9778 | 0.5786 |
|  | rs7076247 | 10 | 18759629 | C | T | -0.2557 | 0.0309 | 1.33E-16 | 0.6114 | -0.012 | 0.0096 | 0.2105998 | 0.6108 |
|  | rs7077127 | 10 | 18717348 | T | G | 0.217 | 0.0334 | 7.95E-11 | 0.2864 | -0.0022 | 0.0103 | 0.8292 | 0.2872 |
|  | rs7100884 | 10 | 18812697 | G | A | 0.2064 | 0.0367 | 1.85E-08 | 0.7845 | 0.001 | 0.0113 | 0.9312001 | 0.7812 |
|  | rs72786085 | 10 | 18713206 | C | G | -0.5309 | 0.0595 | 4.46E-19 | 0.0792 | 0.006 | 0.0169 | 0.7214994 | 0.0848 |
|  | rs72786098 | 10 | 18729855 | A | G | -0.5033 | 0.0883 | 1.18E-08 | 0.0322 | -0.0051 | 0.0255 | 0.8401 | 0.0343 |
|  | rs74593582 | 10 | 18374059 | C | T | 0.7581 | 0.1349 | 1.91E-08 | 0.0154 | -0.0382 | 0.0405 | 0.3446998 | 0.0146 |
|  | rs75699707 | 10 | 18359294 | A | G | -0.5761 | 0.1028 | 2.10E-08 | 0.0264 | -0.002 | 0.0287 | 0.9439 | 0.0268 |
|  | rs7917532 | 10 | 18373902 | C | T | -0.2323 | 0.0304 | 2.01E-14 | 0.5347 | -0.005 | 0.0093 | 0.5936001 | 0.5357 |
|  | rs7923191 | 10 | 18727901 | G | A | 0.369 | 0.0376 | 1.09E-22 | 0.2082 | 0.0072 | 0.0116 | 0.5347995 | 0.205 |
|  | rs982003 | 10 | 18707296 | T | C | -0.2414 | 0.0351 | 6.21E-12 | 0.7568 | 0.0166 | 0.0108 | 0.1260001 | 0.7557 |
| SLC12A2 | rs1351141 | 5 | 127550055 | T | G | 0.2052 | 0.0356 | 8.26E-09 | 0.2341 | -0.009 | 0.0109 | 0.4075998 | 0.2371 |
|  | rs17676242 | 5 | 127589648 | A | G | 0.4156 | 0.0533 | 6.62E-15 | 0.0915 | -0.023 | 0.016 | 0.1519001 | 0.0943 |
|  | rs3101725 | 5 | 127524018 | C | T | 0.1979 | 0.0356 | 2.75E-08 | 0.7583 | -0.0383 | 0.0109 | 0.0004211 | 0.7599 |
|  | rs4836365 | 5 | 127476770 | A | C | 0.3483 | 0.0478 | 3.15E-13 | 0.1129 | -0.019 | 0.0145 | 0.1894999 | 0.1163 |
|  | rs62373688 | 5 | 127352807 | A | T | 0.2742 | 0.0454 | 1.58E-09 | 0.1306 | -0.0156 | 0.0137 | 0.2538002 | 0.1338 |
|  | rs6860245 | 5 | 127367998 | C | G | -0.2112 | 0.0353 | 2.10E-09 | 0.2435 | 0.0386 | 0.0108 | 0.0003304 | 0.2481 |

Supplementary Table S4. Details of the IVs used for MR analysis [causal effect of SBP on KOA (ebi-a-GCST005813)]

| Gene | **SNP** | **Chr** | | **Pos** | **EA** | | **OA** | **Exposure** | | | | **Outcome** | | | |
| --- | --- | --- | --- | --- | --- | --- | --- | --- | --- | --- | --- | --- | --- | --- | --- |
|  |  |  |  |  |  |  |  | **Beta** | **SE** | ***P*-value** | **EAF** | **Beta** | **SE** | ***P*-value** | **EAF** |
| CACNA1D | rs113210396 | 3 | 53612327 | | T | G | | -0.4338 | 0.077 | 1.76E-08 | 0.0451 | 0.118848 | 0.054471 | 0.03078931 | 0.0452055 |
|  | rs114718455 | 3 | 53464055 | | G | A | | 0.5102 | 0.0905 | 1.72E-08 | 0.0336 | 0.00085125 | 0.0638113 | 0.98936001 | 0.0348896 |
|  | rs114987861 | 3 | 53605712 | | A | G | | 0.5289 | 0.0958 | 3.36E-08 | 0.0284 | 0.00397278 | 0.0757548 | 0.95828502 | 0.0262366 |
|  | rs2633731 | 3 | 53738424 | | C | T | | 0.1963 | 0.0309 | 2.21E-10 | 0.6038 | 0.00015328 | 0.0244992 | 0.994952 | 0.594532 |
|  | rs312487 | 3 | 53545622 | | C | T | | -0.2194 | 0.0307 | 9.65E-13 | 0.5217 | 0.0107362 | 0.0241874 | 0.65711442 | 0.521818 |
|  | rs3774472 | 3 | 53638200 | | G | A | | 0.1747 | 0.0303 | 8.21E-09 | 0.4943 | 0.00030292 | 0.0237997 | 0.98984599 | 0.492663 |
|  | rs3821843 | 3 | 53558012 | | A | G | | 0.3373 | 0.0335 | 6.56E-24 | 0.6808 | -0.0071963 | 0.0262147 | 0.78374359 | 0.678193 |
|  | rs62250937 | 3 | 53870318 | | C | T | | -0.3238 | 0.056 | 7.28E-09 | 0.0893 | 0.00703363 | 0.042787 | 0.86950603 | 0.0878809 |
|  | rs7340705 | 3 | 53734443 | | C | T | | 0.2425 | 0.0322 | 4.87E-14 | 0.3268 | 0.00636376 | 0.0257322 | 0.80471408 | 0.319039 |
|  | rs9311502 | 3 | 53560321 | | C | T | | 0.2463 | 0.0355 | 3.87E-12 | 0.2391 | -0.0090287 | 0.027913 | 0.74623051 | 0.237098 |
| CACNB2 | rs10764319 | 10 | 18428415 | | T | C | | 0.2693 | 0.0329 | 2.54E-16 | 0.3044 | 0.00431536 | 0.0262575 | 0.86948001 | 0.293771 |
|  | rs10828399 | 10 | 18553968 | | A | G | | -0.1947 | 0.0302 | 1.10E-10 | 0.5218 | 0.0226577 | 0.023733 | 0.33970505 | 0.504581 |
|  | rs10828452 | 10 | 18592450 | | T | A | | -0.3046 | 0.0388 | 4.20E-15 | 0.207 | 0.0616952 | 0.0297329 | 0.03868566 | 0.208856 |
|  | rs10828542 | 10 | 18627285 | | G | A | | -0.1817 | 0.0311 | 5.18E-09 | 0.3863 | 0.0266542 | 0.0242977 | 0.27290595 | 0.390132 |
|  | rs10828749 | 10 | 18756881 | | A | G | | -0.3658 | 0.0309 | 2.27E-32 | 0.412 | 0.00112803 | 0.0243491 | 0.96309405 | 0.408441 |
|  | rs10828784 | 10 | 18788273 | | G | C | | -0.2021 | 0.0345 | 4.49E-09 | 0.3367 | 0.0130991 | 0.0269106 | 0.62658357 | 0.340725 |
|  | rs11012811 | 10 | 18438456 | | T | G | | 0.3095 | 0.0326 | 2.31E-21 | 0.31 | 0.0161069 | 0.0259284 | 0.53471455 | 0.30272 |
|  | rs11013910 | 10 | 18663370 | | A | G | | 0.2672 | 0.0449 | 2.70E-09 | 0.1306 | -0.0068944 | 0.0354197 | 0.84559109 | 0.133211 |
|  | rs11013938 | 10 | 18669271 | | C | G | | -0.3265 | 0.035 | 1.17E-20 | 0.2554 | 0.05898 | 0.0271214 | 0.03009123 | 0.258141 |
|  | rs11014021 | 10 | 18682233 | | A | T | | -0.3245 | 0.0307 | 4.29E-26 | 0.4182 | 0.00953472 | 0.0243048 | 0.6948835 | 0.415533 |
|  | rs112133583 | 10 | 18695681 | | T | C | | -0.5546 | 0.0973 | 1.18E-08 | 0.0299 | -0.0576649 | 0.0720705 | 0.42089232 | 0.0313326 |
|  | rs112701401 | 10 | 18644811 | | G | C | | -0.5026 | 0.0921 | 4.91E-08 | 0.0305 | -0.043232 | 0.0708886 | 0.54019679 | 0.0311627 |
|  | rs116936375 | 10 | 18737135 | | A | G | | -0.5739 | 0.081 | 1.40E-12 | 0.0405 | -0.0196849 | 0.0637965 | 0.75723382 | 0.0368186 |
|  | rs12258967 | 10 | 18727959 | | G | C | | -0.6327 | 0.0337 | 1.08E-78 | 0.2953 | 0.0141782 | 0.0258771 | 0.58396121 | 0.300587 |
|  | rs12416030 | 10 | 18789075 | | C | T | | 0.2088 | 0.0381 | 4.32E-08 | 0.2031 | -0.0332112 | 0.0304234 | 0.27400974 | 0.204087 |
|  | rs12416052 | 10 | 18789267 | | C | T | | -0.1987 | 0.0311 | 1.59E-10 | 0.4053 | 0.0218976 | 0.0243847 | 0.36936607 | 0.40805 |
|  | rs12765240 | 10 | 18438372 | | T | C | | 0.396 | 0.052 | 2.52E-14 | 0.0932 | 0.036573 | 0.0403464 | 0.36611287 | 0.0928891 |
|  | rs12778700 | 10 | 18385490 | | C | T | | -0.1972 | 0.033 | 2.24E-09 | 0.3323 | -0.0084344 | 0.0254334 | 0.74010442 | 0.343637 |
|  | rs1539680 | 10 | 18502889 | | C | G | | -0.3259 | 0.0375 | 3.37E-18 | 0.7929 | -0.017533 | 0.0293791 | 0.5510538 | 0.797093 |
|  | rs16916922 | 10 | 18467744 | | T | A | | 0.3662 | 0.0433 | 2.86E-17 | 0.1415 | -0.0249115 | 0.0347289 | 0.47238598 | 0.140399 |
|  | rs1757213 | 10 | 18537594 | | G | A | | -0.3084 | 0.0507 | 1.15E-09 | 0.888 | 0.0226288 | 0.036916 | 0.53915669 | 0.877385 |
|  | rs1757225 | 10 | 18516925 | | G | A | | -0.2453 | 0.0325 | 4.29E-14 | 0.6855 | 0.00204865 | 0.0257939 | 0.936651 | 0.692513 |
|  | rs17604757 | 10 | 18442940 | | G | A | | 0.5022 | 0.0606 | 1.12E-16 | 0.0675 | 0.0211177 | 0.048085 | 0.66112107 | 0.0642526 |
|  | rs17610275 | 10 | 18621630 | | G | T | | -0.3868 | 0.0613 | 2.87E-10 | 0.0734 | 0.0770011 | 0.044565 | 0.08600229 | 0.0716243 |
|  | rs1888693 | 10 | 18440444 | | A | G | | 0.3858 | 0.0317 | 4.69E-34 | 0.3449 | -0.0014075 | 0.0250348 | 0.95511508 | 0.343307 |
|  | rs1891392 | 10 | 18336421 | | C | T | | -0.2276 | 0.0334 | 9.02E-12 | 0.7129 | 0.00187027 | 0.0262319 | 0.9431429 | 0.709505 |
|  | rs1998822 | 10 | 18755664 | | G | A | | 0.1958 | 0.0343 | 1.15E-08 | 0.2766 | -0.0061453 | 0.0263491 | 0.81553593 | 0.281549 |
|  | rs4748444 | 10 | 18494482 | | C | T | | -0.1939 | 0.0327 | 3.13E-09 | 0.3363 | -0.0127938 | 0.0249651 | 0.60817141 | 0.340593 |
|  | rs4748472 | 10 | 18776197 | | T | C | | 0.3161 | 0.0319 | 4.04E-23 | 0.6558 | -0.0214037 | 0.0251281 | 0.39464536 | 0.658167 |
|  | rs61278674 | 10 | 18481737 | | G | A | | 0.3298 | 0.054 | 1.03E-09 | 0.0938 | -0.0061458 | 0.0412537 | 0.88150991 | 0.0985437 |
|  | rs6482184 | 10 | 18403118 | | C | T | | 0.1949 | 0.0316 | 6.65E-10 | 0.3795 | 0.016042 | 0.0247581 | 0.51718509 | 0.370722 |
|  | rs67214975 | 10 | 18727251 | | A | C | | -0.4144 | 0.0307 | 1.42E-41 | 0.4563 | 0.0227101 | 0.0239076 | 0.34222994 | 0.459057 |
|  | rs7074171 | 10 | 18676897 | | G | A | | 0.3061 | 0.0314 | 1.76E-22 | 0.5795 | -0.0043137 | 0.0246805 | 0.86126203 | 0.575119 |
|  | rs7076247 | 10 | 18759629 | | C | T | | -0.2557 | 0.0309 | 1.33E-16 | 0.6114 | -0.004241 | 0.0244092 | 0.86207907 | 0.60766 |
|  | rs7077127 | 10 | 18717348 | | T | G | | 0.217 | 0.0334 | 7.95E-11 | 0.2864 | -0.0031896 | 0.0264658 | 0.9039939 | 0.286558 |
|  | rs7100884 | 10 | 18812697 | | G | A | | 0.2064 | 0.0367 | 1.85E-08 | 0.7845 | 0.00229385 | 0.028795 | 0.93647201 | 0.782063 |
|  | rs72786085 | 10 | 18713206 | | C | G | | -0.5309 | 0.0595 | 4.46E-19 | 0.0792 | -0.0456116 | 0.0432894 | 0.29014093 | 0.0888826 |
|  | rs72786098 | 10 | 18729855 | | A | G | | -0.5033 | 0.0883 | 1.18E-08 | 0.0322 | -0.045857 | 0.0649941 | 0.47856397 | 0.0356444 |
|  | rs74593582 | 10 | 18374059 | | C | T | | 0.7581 | 0.1349 | 1.91E-08 | 0.0154 | -0.060885 | 0.0984131 | 0.53354002 | 0.0164154 |
|  | rs75699707 | 10 | 18359294 | | A | G | | -0.5761 | 0.1028 | 2.10E-08 | 0.0264 | 0.0693326 | 0.0712209 | 0.33338035 | 0.026922 |
|  | rs7917532 | 10 | 18373902 | | C | T | | -0.2323 | 0.0304 | 2.01E-14 | 0.5347 | -0.0103272 | 0.0240497 | 0.66764869 | 0.538263 |
|  | rs7923191 | 10 | 18727901 | | G | A | | 0.369 | 0.0376 | 1.09E-22 | 0.2082 | 0.00021612 | 0.0297798 | 0.994269 | 0.203793 |
|  | rs982003 | 10 | 18707296 | | T | C | | -0.2414 | 0.0351 | 6.21E-12 | 0.7568 | 0.0287128 | 0.0277579 | 0.30021128 | 0.754684 |
| SLC12A2 | rs1351141 | 5 | 127550055 | | T | G | | 0.2052 | 0.0356 | 8.26E-09 | 0.2341 | -0.0232488 | 0.0281008 | 0.40745627 | 0.237233 |
|  | rs17676242 | 5 | 127589648 | | A | G | | 0.4156 | 0.0533 | 6.62E-15 | 0.0915 | -0.0261166 | 0.0414888 | 0.52813141 | 0.09454 |
|  | rs3101725 | 5 | 127524018 | | C | T | | 0.1979 | 0.0356 | 2.75E-08 | 0.7583 | -0.0169947 | 0.0275451 | 0.53760958 | 0.760039 |
|  | rs4836365 | 5 | 127476770 | | A | C | | 0.3483 | 0.0478 | 3.15E-13 | 0.1129 | -0.0427435 | 0.0376709 | 0.2549001 | 0.114869 |
|  | rs62373688 | 5 | 127352807 | | A | T | | 0.2742 | 0.0454 | 1.58E-09 | 0.1306 | -0.0458959 | 0.035573 | 0.1954029 | 0.132879 |
|  | rs6860245 | 5 | 127367998 | | C | G | | -0.2112 | 0.0353 | 2.10E-09 | 0.2435 | 0.017072 | 0.0272838 | 0.53185347 | 0.247482 |
